# Establishment of a voluntary electronic *Chlamydia trachomatis* laboratory surveillance system in Germany, 2008 to 2014

**DOI:** 10.2807/1560-7917.ES.2017.22.6.30459

**Published:** 2017-02-09

**Authors:** Sandra Dudareva-Vizule, Karin Haar, Andrea Sailer, Klaus Jansen, Osamah Hamouda, Hilmar Wisplinghoff, Carsten Tiemann, Eberhard Pape, Viviane Bremer, Chlamydia trachomatis laboratory sentinel team

**Affiliations:** 1 Department for Infectious Disease Epidemiology, Robert Koch Institute, Berlin, Germany; 2 Charité University Medicine, Berlin, Germany; 3 Wisplinghoff Laboratories, Cologne, Germany; 4 Institute for Medical Microbiology, University of Cologne, Cologne, Germany; 5 Institute for Microbiology, University Witten/Herdecke, Witten, Germany; 6 Labor Krone, Bad Salzuflen, Germany; 7 The Chlamydia trachomatis laboratory sentinel team is listed at the end of the article

**Keywords:** Chlamydia trachomatis, sexually transmitted infections, Germany, surveillance, laboratory surveillance, screening, sentinel

## Abstract

*Chlamydia trachomatis* (CT) infections are not reportable in Germany and limited data on prevalence are available. CT screening has been offered free of charge to pregnant women since 1995 and to all women under 25 years since 2008. For symptomatic women and men, diagnostic testing is covered by statutory health insurance. We describe the establishment of a nationwide, laboratory-based, voluntary sentinel that electronically collects information on all performed CT tests with test results, test reason and patient information. The sentinel represents one third of all performed CT tests in Germany. In the period from 2008 to 2014, 3,877,588 CT tests were reported, 93% in women. Women aged 20–24 years and men aged 25–29 years were the most frequently tested age groups. The overall proportion of positive tests (PPT) among women was 3.9% and among men 11.0%. The highest PPT among women was in the age groups 15–19 (6.8%) and 20–24 years (5.9%), and among men in the age groups 20–24 (19.2%), 15–19 (15.4%) and 25–29 years (14.8%). The PPT for CT was high among women and men younger than 25 years. Prevention is urgently needed. Monitoring of CT infection in Germany should be continued.

## Introduction

Infections with *Chlamydia trachomatis* (CT) rank among the most frequent sexually transmitted infections (STI) in Europe and worldwide [1,2]. According to European data, the most affected age groups are women aged 15–24 years and men aged 20–24 years [2,3].The CT infection may be asymptomatic and can, if not detected and treated, result in complications such as pelvic inflammatory disease, chronic abdominal pain, ectopic pregnancy, tubal sterility, a higher risk of adverse pregnancy outcomes for women and of epididymitis for men [3-10]. Evidence whether CT screening can prevent these complications is, however, controversial [3].

CT infections are not reportable in Germany, except for one federal state (Saxony), where we observed a continuous increase from 40.8 reported CT infections per 100,000 population in 2004 to 101.0 in 2012 [11]. However, only detected infections are reported. The true incidence in the population might be higher owing to the large proportion of asymptomatic infections that might remain undetected. In population-wide studies in Germany performed between 2003 and 2006, we observed a prevalence of up to 4.5% among women aged 17–19 years and 4.9% among men aged 25–29 years [12-14]. Between 2003 and 2009, data on CT were collected through the STI sentinel surveillance system from 247 sites (mainly local municipality counselling centres for STI, followed by STI outpatient clinics, general practitioners and other specialists) situated all over Germany but not representative of the general population in Germany. CT was the most frequently diagnosed STI, with a positivity of 6.0% among performed tests [15-17]. Sixty-seven per cent of the diagnosed CT infections were among women, many of them working as sex workers who attended the free-of-charge local municipality counselling clinics. The median age of infected women was 25 years and of men 31 years [15,16].

Health insurance in Germany is compulsory and individuals are covered either by statutory health insurance (ca 90%) or private health insurance. Private health insurance is available only to some segments of the population [18].

Patients in Germany can freely choose their medical practitioner, i.e. not based on place of residence. Laboratories do not have a defined catchment area, thus, there are laboratories serving only surrounding areas as well as laboratories receiving samples from all over Germany.

Since 1995, opportunistic CT screening for pregnant women with statutory insurance has been in place, and in 2008, yearly CT screening for sexually active women under the age of 25 years with statutory insurance, as well as a CT test before planned abortion, was introduced in Germany [19]. Up until now, there have been no CT screening programmes for men. Health insurance companies can reimburse men and women for the costs of testing if they report specific symptoms or unspecific symptoms together with risk behaviour or if a sex partner has been tested positive for CT (diagnostic testing). Otherwise, the CT test can be requested and paid by the patient.

CT has been classified in the highest priority group of pathogens in Germany [20]. However, data on the proportion of positive tests (PPT) in different age groups and regions are limited. Furthermore, there are no data on the frequency of the different test indications for CT in women and on the coverage of the screening programme for women younger than 25 years. Except for Saxony, there is no information on the CT infection trend over time.

To close this knowledge gap, we introduced a new laboratory-based CT surveillance system, the ‘CT laboratory sentinel’ in Germany in 2010. The aim of the CT laboratory sentinel was to monitor CT testing data and infections in Germany and to evaluate the newly introduced CT screening for women under 25 years of age, in order to develop public health recommendations for targeted prevention measures.

Before the laboratory-based CT-surveillance was set up, all laboratories testing for CT in Germany were mapped [21]. Of 1,504 contacted facilities, 725 (48%) responded to a questionnaire; 143 reported that they performed CT diagnostics and of those 143, 60 reported that they would be interested in reporting data [21].

In this paper, we report on how the CT laboratory sentinel was established and present the first results.

## Methods

### Establishment of the *Chlamydia trachomatis* laboratory sentinel

In September 2010, we started implementing a voluntary laboratory-based sentinel system in Germany for electronic and, where possible, automated collection of information that is routinely available in laboratories on CT tests. Mapping of the laboratories performing CT diagnostics [21] provided us with a list of laboratories that expressed interest in participation and with information on the number of CT tests per quarter and catchment area. We recruited laboratories based on the number of performed CT tests and on the size of the catchment area. Our aim was to recruit laboratories performing many CT tests and to reach equally good geographical distribution in each federal state. After review of the geographical distribution and coverage in our sample, we decided to recruit additional laboratories with catchment areas from underrepresented regions.

Through the CT laboratory sentinel, we collected retrospective (back to 2008) and continuous data on the performed CT tests up to 31 December 2014. Data were reported on a quarterly basis. The laboratories indicated that not all variables could be selected from their data systems or the selection would be very time consuming. To keep the effort reasonable, we defined a standard common set of mandatory and optional variables. Mandatory variables were sample and patient identification number, date of laboratory testing, test result, sex, and year of birth. Optional variables included date of sampling, the first three digits of the standard five-digit postal code of the patient, the first three digits of the postal code of the submitting medical practitioner, month of birth, reason for testing or billing codes (used for invoicing health insurance), pregnancy status, tested material, health insurance status (statutory or private) and method of testing.

If the three-digit postal code of the patient was not available, we used the three-digit postal code of the submitting medical practitioner. We generated information on the test reason from the reported reason for testing or respective billing codes. Samples from female patients, who were tested because of symptoms or suspicion of infection, were categorised as ‘Diagnostic testing’. Samples from female patients who were tested during pregnancy or before a planned abortion were categorised as ‘Screening in pregnancy’. Women who were screened as part of the screening for under 25 years of age were categorised as ‘Screening for women under 25’.

On the basis of the year of birth, we calculated patient age at the time of testing. Laboratories reported the CT tests for the complete time period, or less if reporting for the complete time period was not possible.

Data were transmitted electronically. The data were sent to us either via email as extensible markup language (.XML) files, comma-separated values (.CSV) or Excel spreadsheet (.XLS) files or in XML format via secure sockets layer (SSL)-encrypted Internet connection to a web service. After performing predefined automated plausibility checks, the received data were combined in a structured query language (SQL) database. Unplausible variables were set to missing. The sample and patient numbers were MD5-encrypted and transmitted as 32-digit hash codes. Decryption of this code was not possible. If patients were tested more than once at the same laboratory, the 32-digit hash code enabled us to assign data from several samples over time to one patient. However, samples from the same patient tested in different laboratories could not be assigned to the same patient. If laboratories used different input data (for example, surname and date of birth in one quarter and name plus surname and date of birth in the subsequent quarter) to generate the 32-digit hash codes, we were not able to trace those patient numbers over time. In order to understand if person-related analysis, such as testing frequency and time intervals between tests, is possible for this way of data collection, we proved the traceability of the patient identification numbers by laboratory over time.

There was no financial compensation for laboratories to participate in the study. The data collection protocol was confirmed by the data protection officer at the Robert Koch Institute, Berlin. Additional approval from an ethics committee was not deemed necessary, as no patient-identifying data were collected.

### Data analysis

We analysed all CT tests available for the time period between 1 January 2008 and 31 December 2014. For the reported CT tests, we calculated counts and proportions of the available and missing variables. We calculated the duration of the reporting period by laboratory as well as counts and proportions of the CT tests by laboratory.

We defined coverage as the proportion of CT tests from individuals with statutory insurance collected through the sentinel among all CT tests from individuals with statutory insurance. The National Association of Statutory Health Insurance provided us with data on all performed CT tests from individuals with statutory insurance for the years 2011 and 2012. We are not able to individually link patients or tests in the two data sources. Instead, we first calculated the proportion of individuals with statutory insurance among the CT tests with available information on health insurance status. Then, we extrapolated this proportion to all CT tests collected within the laboratory sentinel in the years 2011 and 2012 and calculated the total number of CT tests from individuals with statutory insurance. Finally, we assessed the coverage of the laboratory sentinel by comparing the total number of CT tests from persons with statutory insurance in Germany and from persons with statutory insurance collected in the CT laboratory sentinel. We assumed that the coverage of CT tests for privately insured persons was similar.

The geographical distribution of the reported CT tests based on the postal codes was described as the number of CT tests per 100,000 population by federal state in Germany.

We described CT tests in the laboratory sentinel and the PPT by age group, sex, reason for testing (diagnostic testing because of symptoms, screening in pregnancy or screening for women under 25 years of age) and tested material (for men).

## Results

### Participating laboratories and collected data

Of the 60 laboratories selected for recruitment, 24 agreed to participate and have been reporting data to the CT laboratory sentinel. The reasons for refusing to participate were: data selection in the requested format was not possible (n = 10), too much effort was required (n = 12), CT samples were forwarded to a partner/alliance laboratory or organisational changes (n = 7), refusal without a specific reason (n = 4), other reasons (n = 3). Three of the laboratories refusing to participate were large laboratories with a nationwide catchment area. Currently, two laboratories are reporting data by using the web service; three send the data as XML, five as CSV and 14 as Excel spreadsheets via email.

By 24 November 2015, a total of 3,877,588 CT tests had been reported for the period from 1 January 2008 to 31 December 2014. A total of 15 laboratories have reported data for each quarter of the entire study period. A further nine laboratories have reported data for a minimum of 1 month and a maximum of 4 years and 7 months (Table 1).

**Table 1:** Number of reported *Chlamydia trachomatis* tests, proportion of tests in women, data reporting period, catchment area and patient traceability period by laboratory, Germany 2008–2014 (n = 3,877,588)

Laboratory	Reported CT tests n^a^	Tests in Women %	Reporting period (number of CT tests)							Patient traceability period
			2008	2009	2010	2011	2012	2013	2014	
1	1,629,040	98.0	78,770	183,085	211,571	244,480	268,823	309,423	332,888	Partly
2	450,368	93.0	49,184	59,628	66,939	63,678	63,383	70,182	77,374	Complete
3	342,929	91.1	41,451	51,327	51,190	50,430	48,649	50,946	48,936	Complete
4	268,248	86.2	15,821	33,004	34,360	39,379	47,893	47,990	49,801	Partly
5	200,204	90.4	15,366	27,885	29,839	29,859	31,142	33,842	32,271	Complete
6	132,726	92.4	ND	ND	ND	ND	ND	282	132,444	Partly
7	126,170	96.6	2,609	19,884	19,836	20,892	21,919	21,663	19,367	Complete
8	95,022	74.0	8,964	10,618	11,548	12,336	14,261	15,805	21,490	Complete
9	92,824	80.2	12,162	12,882	11,529	11,746	11,998	15,684	16,823	Partly
10	83,715	92.3	8,338	11,924	12,025	12,973	12,282	13,236	12,937	Complete
11	64,806	94.9	ND	ND	ND	ND	ND	29,906	34,900	Patient number missing
12	64,133	96.6	7,705	8,449	8,894	8,937	9,482	10,118	10,548	Complete
13	59,098	99.9	5,834	14,592	15,013	14,583	9,076			Complete
14	51,755	90.7	6,524	7,371	7,128	7,857	7,715	7,460	7,700	Complete
15	49,839	84.1	3,219	7,594	7,452	7,533	7,162	7,926	8,953	Complete
16	45,708	94.7	ND	ND	ND	ND	15,816	14,951	14,941	Partly
17	39,969	70.3	ND	ND	ND	ND	64	20,017	19,888	Complete
18	29,573	90.9	ND	ND	ND	7,849	7,296	7,251	7,177	Partly
19	28,636	32.9	3,569	3,541	3,549	3,849	4,573	4,782	4,773	Complete
20	12,398	63.9	1,587	1,437	1,411	1,769	1,822	2,065	2,307	Complete
21	8,105	66.8	766	895	995	1,198	1,053	1,260	1,938	Complete
22	1,590	69.7	ND	ND	ND	422	397	391	380	Partly
23	564	97.2	ND	ND	ND	554	10	ND	ND	Patient number missing
24	168	92.3	ND	ND	ND	ND	168	ND	ND	Complete
**Total**	**3,877,588**	**92.8**	**261,869**	**454,116**	**493,279**	**540,324**	**584,984**	**685,180**	**857,836**	

CT: Chlamydia trachomatis; ND: no data reported.

^a^ Laboratories sorted by number of reported CT tests.

Information on the mandatory variables was missing in less than 1% of all reported CT tests and on optional variables between 13% and 80% (Table 2). Patient number was consistently coded and therefore traceable over the entire reporting time in 15 laboratories, consistently coded only for part of the time in seven laboratories, and two laboratories did not report patient identification numbers (Table 1).

**Table 2:** Number of *Chlamydia trachomatis* records with information on collected variables, and number and proportion of records with unknown, missing or implausible information, Germany, 2008–2014 (n = 3,877,588)

Variable	Type of variable	Available	Unknown, missing or unplausible	
		n	n	%
Sample number	Mandatory	3,877,588	0	0.0
Patient number	Mandatory	3,877,588	0	0.0
Test result	Mandatory	3,856,972	20,616	0.5
Sex	Mandatory	3,855,455	22,133	0.6
Year of birth	Mandatory	3,859,684	17,904	0.5
Month of birth	Optional	3,313,251	564,337	17.0
Three-digit postal code^a^	Optional	3,220,557	657,031	20.4
Date of sampling	Optional	776,349	3,101,239	80.0
Test reason	Optional	3,496,011	381,577	9.8
Pregnancy status	Optional	2,954,620	922,968	23.8
Tested material	Optional	3,378,347	499,241	12.9
Method of testing	Optional	2,126,643	1,750,945	45.2
Health insurance status	Optional	1,207,750	2,669,838	68.9

^a^ For 3,097,980 records (96.2%), patient three-digit postal codes were reported, and for 122,577 records (3.8%), postal codes of practitioners were reported.

### Coverage

In total, 91.1% and 78.1% of CT tests with information on health insurance were attributable to, respectively, the women and men with statutory health insurance. We estimated that 34.3% of all CT tests performed among statutorily insured persons in Germany were reported to the CT laboratory sentinel. These estimates varied by federal state from 4.4% in Baden-Wurttemberg to 60.9% in Thuringia (Table 3). The coverage was 34.6 for CT tests among women and 28.7% for CT tests among men (Table 3).

**Table 3:** Proportion of *Chlamydia trachomatis* tests from men and women with statutory health insurance (n = 2,964,346), collected in the *Chlamydia trachomatis* laboratory sentinel, by federal state, Germany, 2011–12 (n = 1,016,231)

**Federal state (total population^a^)**	Proportion of CT tests collected through the sentinel (%)			
	**Women**	**Men**	**Unknown**	**Total**
Baden-Wurttemberg (n = 10,786,227)	4.3	6.3	18.4	4.4
Bavaria (n = 12,595,891)	29.6	16.1	19.2	28.9
Berlin (n = 3,501,872)	57.9	53.2	177	57.3
Brandenburg (n = 2,495,635)	37.2	8.8	3.5	35.7
Bremen (n = 661,301)	12.2	1.9	0.0	11.5
Hamburg (n = 1,798,836)	15.3	2.4	0.7	13.5
Hesse (n = 6,092,126)	19.7	22.9	44.6	20.0
Lower Saxony (n = 7,913,502)	24.7	12.2	1.4	23.8
Mecklenburg-Western Pomerania (n = 1,634,734)	53.9	25.9	17.7	52.4
North Rhine-Westphalia (n = 17,841,956)	39.6	34.9	66.4	39.4
Rhineland-Palatinate (n = 3,999,117)	29.8	18.9	1170	29.5
Saarland (n = 1,013,352)	19.6	8.8	90.5	19.0
Saxony (n = 4,137,051)	27.7	35.4	49.0	28.4
Saxony-Anhalt (n = 2,313,280)	36.1	6.9	1.4	33.8
Schleswig-Holstein (n = 2,837,641)	22.6	3.3	5.5	21.1
Thuringia (n = 2,221,222)	60.1	82.6	41.5	60.9
**Total**	**34.6**	**28.7**	**177.3**	**34.3**

CT: Chlamydia trachomatis.

^a^ Population by federal state in 2011 (Source: German Federal Statistical Office).

### Regional distribution

**Figure 1:**
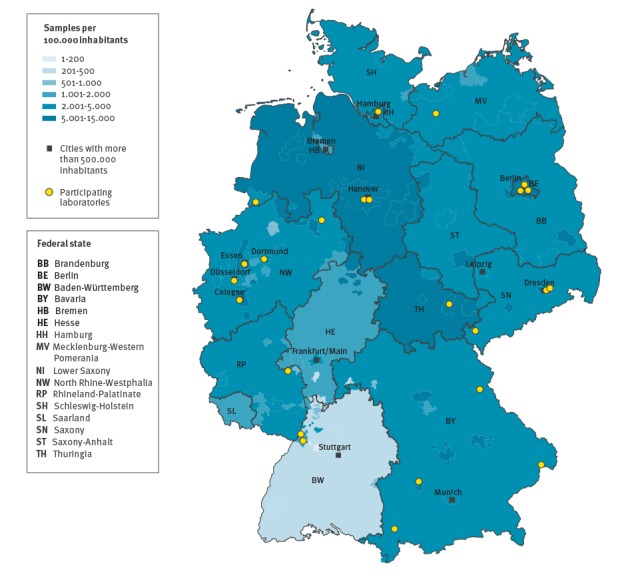
Number of reported *Chlamydia trachomatis* tests per 100,000, by federal state, Germany, 2008–14 (n = 3,220,628)

The number of reported CT tests with information on the three-digit postal code for the entire period per 100,000 of the population varied by region between 141 and 14,901 (Figure 1). Based on information on the catchment areas provided from laboratories that did not report information on postal code, ca 50% of CT tests with missing postal codes would be from Saxony, around 40% from the western part of the country (Bremen, Lower Saxony, North Rhine-Westphalia, Hesse, Rhineland-Palatinate, Saarland and Baden-Württemberg) and the rest from Berlin.

### CT testing

**Figure 2:**
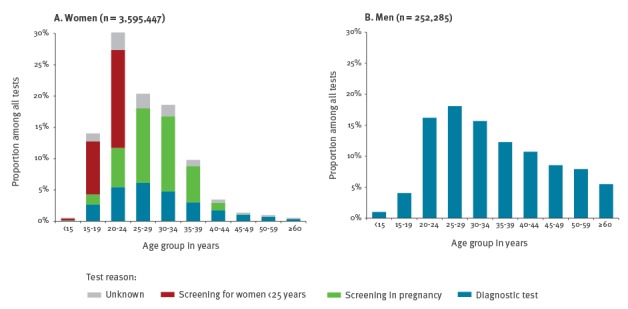
Proportion of reported *Chlamydia trachomatis* tests by age group, test reason and sex, Germany, 2008–14

Of the total of 3,877,588 reported CT tests for the period 2008 to 2014, 92.8% (3,599,821) were done in women and 6.6% (255,634) in men. Among women with information on age (3,595,447), the most frequently tested age groups were women aged 20–24 years, and among men (252,285) those aged 25–29 years, followed by those aged 20–24 years and 30–34 years. The proportion of CT tests by age group among men and women are reported in Figure 2.

#### Reason for testing in women

Among CT tests in women with information on the reason for testing, 41.9% were attributable to screening in pregnancy, 26.9% to screening of women under 25 years of age and 28.7% to diagnostic tests (Figure 2).

#### Tested material in men

Among CT tests in men with information on tested specimen, 49.0% were unspecified swabs, 32.5% urine, 5.3% urethral, 3.1% rectal and 1.9% pharyngeal swabs. In 8.2% of tests, other materials were tested.

#### Proportion of positive tests

Among tests with valid test results (n = 3,827,792), 3.9% (95% confidence interval (CI): 3.9–4.0) of tests among women and 11.0% (95% CI: 10.9–11.2) of tests among men were positive. PPT varied by federal state from 3.0% in Saarland to 6.8% in Mecklenburg-Western Pomerania among women and 9.0% in Saarland to 17.0% in Mecklenburg-Western Pomerania among men.

**Figure 3:**
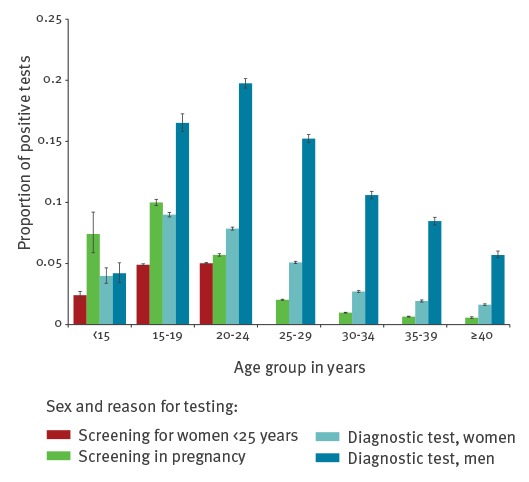
Proportion of positive *Chlamydia trachomatis* tests with 95% confidence intervals, by sex, age group and test reason, Germany, 2008–14, among women (n = 3,577,935) and men (n = 249,857)

The PPT among women differed by reason for testing and age (Figure 3). Overall, the highest PPT was observed among women aged 15–19 years and 20–24 years (Figure 3). The PPT when screening women under 25 years was 4.9% in 15–19 and 5.0% in 20–24 year-olds. While screening tests in pregnancy and diagnostic testing revealed, respectively, a PPT of 10.0% and 9.0% among 15–19 year-olds and 5.7% and 7.9% among 20–24 year-olds, PPT among pregnant women decreased to 2.0% among women 25–29 years of age and was < 1% in those 30 years and older. The PPT in diagnostic tests also decreased with increasing age (Figure 3).

Among men, the highest PPT was observed among the age groups 20–24 years (19.2%), 15–19 years (15.4%) and 25–29 years (14.8%). The PPT among women and men decreased with age (Figure 3).

**Figure 4:**
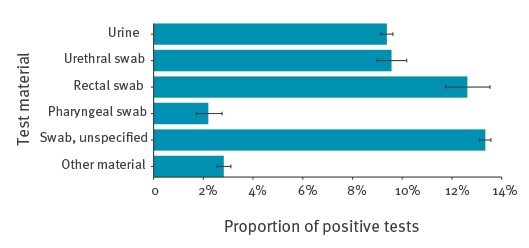
Proportion of positive *Chlamydia trachomatis* tests with 95% confidence intervals among men, by tested material, Germany, 2008–14 (n = 174,346)

Among men, the PPT was higher in rectal (12.3%) and in unspecified swabs (13.4%) (Figure 4).

## Discussion

We established a CT laboratory sentinel in Germany that electronically collects data that are routinely available in laboratories on performed CT tests; the CT laboratory sentinel serves as a surveillance system. In the period from 2008 to 2014, we reached good coverage and collected a large number of samples representing one third of all performed CT tests in Germany, together with epidemiological information and data on testing. In total, 24 laboratories reported data on a voluntary basis; for the majority of the data, we had information for a complete time period (January 2008 to December 2014). Completeness of the five mandatory variables was more than 99%, while completeness of the eight optional variables varied by laboratory and variable.

We estimate that we have collected 34% of all CT tests of individuals with statutory health insurance in the CT laboratory sentinel. This was possible because we were able to recruit some very large laboratories. Although this estimate is based on data from 2011 and 2012, we assume that we have reached at least the same coverage in the following years 2013 and 2014. We also assume that the coverage of CT tests from individuals with private health insurance was similar. The coverage was slightly better for statutorily insured women than men. The reason for this is unclear. One possible explanation may be that statutorily insured men are being tested at specialist HIV centres or at centres targeting men who have sex with men (MSM), and that these centres might be cooperating with local laboratories not included in the sentinel.

We were able to collect data from samples from all over Germany. Baden-Wurttemberg contributed the lowest number of reported CT tests per 100,000 population and also reached the lowest coverage compared with the other federal states. A substantial proportion of the CT tests with missing information on postal codes was reported from one laboratory with a catchment area in Baden-Wurttemberg, Hesse and Rhineland-Palatinate. We therefore assume that the geographical distribution of tested persons in these federal states or neighbouring areas is better than that estimated based on the postal codes. To obtain better regional data and better coverage of the CT tests from men, we are recruiting further laboratories for participation. An update of the laboratory mapping exercise would be desirable to indicate further potential laboratories covering Baden-Wurttemberg that were not reached in the first mapping.

In several laboratories, the number of performed CT test has increased over the years. Based on information provided from laboratories, a substantial part of the observed increase can be attributed to merging or expansion of the laboratories. However, we are not able to quantify this. We believe that there has been a real increase in CT testing activity in Germany. Further analysis of the statutory insurance registry can clarify if the number of performed CT tests has risen since 2008.

The majority of the reported CT tests were from women, as CT screening is offered to women under 25 years of age and pregnant women. Women aged 20–24 years were by far the most frequently tested age group, followed by women aged 25–34 years. Men aged 20–34 years were most frequently tested compared with other age groups. We also observed the highest PPT in age groups with the highest test frequency. PPT among both men and women was high among tests from younger people and decreased with age. In order to analyse the PPT variation by region further, sociodemographic information is necessary.

We observed the highest PPT among women and men aged 15–24 years, which is similar to several population-based studies in Europe [12-14,22-28]. National chlamydia testing data with information on denominator from England and Norway report PPT of, respectively, 7.8% and 11.5% among 15–24 year-old women and of 10.0% and 17.1% among 15–24 year-old men [27,28]. Opportunities for testing free of charge, especially for men, are scarce in Germany, comparison with England and Norway [27,28]. This impacts testing rates, the groups tested and the PPT.

The PPT was high among very young women screened during pregnancy (these data include also CT tests before abortion). This might be explained by a young age at first sexual intercourse, which several studies have linked to having more partners, more diverse sexual experiences, less frequent use of condoms, and increased risk for bacterial STI, pregnancy and abortion [29]. The PPT among CT tests in pregnancy decreased with increasing age and was less than 1% among women older than 30 years. Our data suggest that it is more rational to screen younger pregnant women, especially those under 25 years of age, than older ones. Furthermore, it is likely that with the given PPT in older pregnant women, some tests may be false positive and will lead to unnecessary treatment. With the current data collected in the laboratory sentinel, we cannot determine what proportion of positive CT tests can be explained by risk behaviour, such as new or multiple sexual partners, other STI or history of sex work. Testing groups with higher prevalence is more effective in terms of detection rate. Age- and risk behaviour-indicated screening in pregnancy in Germany instead of screening of all pregnant women should be further discussed. A cost–benefit analysis taking into account estimates of age-specific adverse health outcomes in pregnancy due to chlamydia infection would facilitate these discussions.

PPT was higher in men than in women also when comparing only diagnostic CT tests. This was not unexpected, as we only reported on CT tests performed among men presenting with symptoms. The PPT in rectal swabs compared with urethral samples was high. Therefore we believe that a substantial proportion of positive CT tests among men might be attributable to MSM. Although almost half of the samples tested were unspecified swabs, we believe based on the PPT that a substantial proportion can be attributable to rectal swabs. Among MSM screened for STI in Germany, a CT prevalence of 9.4% (95% CI: 7.1–12.0) has been previously reported [30].

The majority of countries in the European Union and European Economic Area have a system for reporting and monitoring diagnosed CT cases at the population level [31]. These are however limited to infections that have been diagnosed and reported. The CT detection rates are influenced by populations tested and testing volume [31]. The CT laboratory sentinel provides information on both positive and negative test results which allows us to calculate the PPT and monitor it over time.

The limitations of this study are that the laboratories did not have an equal chance to be included in the sentinel, as we were selecting laboratories based on the interest to participate, number of performed CT tests and catchment area. There may be other large laboratories that were not reached in the mapping phase [21] and thus not considered for the laboratory sentinel. Although we evaluated our data for coverage at least once per year and selected for recruitment additional laboratories with catchment areas in regions underrepresented in the sentinel, we could not obtain an even coverage in all regions. Few laboratories reported the optional variables, which could have resulted in a selection bias in these data. However, owing to the large number of reported CT tests, analyses describing these variables are still possible. Efforts are continuing to improve completeness of the optional variables. We are unable to collect more detailed epidemiological information such as route of transmission and symptoms through the CT laboratory sentinel. Usually, laboratories in Germany have only very limited epidemiological information and there is no legal basis to collect these data. Laboratories that have more information need to treat this information confidentially.

## Conclusion

The implementation of our CT laboratory sentinel has shown that it is feasible in Germany to collect, electronically and continuously, readily available data from laboratories with a reasonable effort that can for now be used instead of mandatory surveillance. We managed to collect a large amount of data from all regions in Germany that represented around one third of all performed CT tests. In contrast to mandatory surveillance, the CT laboratory sentinel collects information on all performed CT tests which allows analysis of PPT over time. In addition, regularly conducted population-based prevalence surveys, although costly, could help determine the true prevalence of CT infection in the population and evaluate prevention strategies.

A large PPT among young men and women and low awareness of CT in Germany [32] support the need for further prevention efforts. The CT laboratory sentinel should continue to collect data and expand the base of participating laboratories in order to monitor and describe CT infection in Germany and guide public health strategies. The participating laboratories should be continuously evaluated and the coverage and representation of different groups tested should be improved.

## Notes

**The *Chlamydia trachomatis* laboratory sentinel team:** Michael Baier, Eberhard Straube (Institut für Medizinische Mikrobiologie, Universitätsklinikum Jena), Armin Baillot (Niedersächsisches Landesgesundheitsamt, Hannover), Patricia Bartsch (MVZ Dr Eberhard & Partner, Dortmund), Thomas Brüning (LADR GmbH MVZ Nord-West, Schüttorf), Josef Cremer (Medizinisch-Diagnostisches Labor Kempten, Kempten), Helga Dallügge-Tamm, Arndt Gröning (amedes MVZ wagnerstibbe, Hannover), Stephan Eicke (Labor Dr Eicke, Berlin), Dagmar Emrich (MVZ Labor 28 GmbH, Berlin), Gundula Fritsche (Labor MVZ Westmecklenburg, Schwerin), Rosi Gjavotchanoff (Labor Alfredstraße, Essen), Peter Gohl (Bioscientia Institut für Medizinische Diagnostik GmbH, Ingelheim), Matthias Götzrath, Axel Meye (Medizinisches Labor Ostsachsen MVZ GbR, Dresden), Ingrid Ehrhard, Beate Köpke (Landesuntersuchungsanstalt Sachsen, Dresden), Birgit Henrich (Institut für Medizinische Mikrobiologie und Krankenhaushygiene, Universitätsklinikum Düsseldorf), Caroline Kastilan (MVZ Labor Diagnostik Karlsruhe GmbH, Karlsruhe), Susanne Lehmann (Diagnosticum, Plauen), Anneliese Märzacker (Labor Schottdorf MVZ GmbH, Augsburg), Bernhard Miller (Labor PD Dr Volkmann und Kollegen GbR, Karlsruhe), Gerrit Mohrmann (Labor Lademannbogen MVZ GmbH, Hamburg), Christian Pache (MVZ Dr Engelschalk, Dr Schubach, Dr Wiegel und Kollegen, Passau), Roland Pfüller (MDI Laboratorien GmbH, Berlin), Thomas Müller, Christian Aepinus – (synlab MVZ Weiden GmbH, Weiden).
